# Co-Development of a Web-Based Hub (eSocial-hub) to Combat Social Isolation and Loneliness in Francophone and Anglophone Older People in the Linguistic Minority Context (Quebec, Manitoba, and New Brunswick): Protocol for a Mixed Methods Interventional Study

**DOI:** 10.2196/30802

**Published:** 2021-09-15

**Authors:** Idrissa Beogo, Jean Ramdé, Eric Nguemeleu Tchouaket, Drissa Sia, Nebila Jean-Claude Bationo, Stephanie Collin, Abdoulaye Anne, Marie-Pierre Gagnon

**Affiliations:** 1 École des sciences infirmières Faculté des sciences de la santé Université d'Ottawa Ottawa, ON Canada; 2 College of Nursing Rady Faculty of Health Sciences University of Manitoba Winnipeg, MB Canada; 3 Département des fondements et pratiques en éducation Faculté des sciences de l’éducation Université Laval Québec, QC Canada; 4 Département des sciences infirmières Université du Québec en Outaouais Campus de Saint-Jérôme Campus de Saint-Jérôme, QC Canada; 5 École des hautes études publiques Université de Moncton Campus de Moncton Moncton, NB Canada; 6 Faculté des sciences infirmières Université Laval Québec, QC Canada

**Keywords:** older people, nursing facility, nursing home, long-term care home, linguistic minority, digital health, COVID-19, social isolation, loneliness, older adults, development, isolation, minority, community

## Abstract

**Background:**

The first wave of the COVID-19 pandemic has severely hit Canadian nursing facilities (81% of deaths). To this toll, public health measures (eg, visitation restriction) have subsequently deepened the social isolation and loneliness of residents in nursing facilities (NFs), especially those in linguistic minority settings: Anglophone institutions in Quebec and Francophone institutions outside Quebec. However, very few COVID-19 initiatives targeting these populations specifically have been documented. Given the limited number of NFs serving linguistic minorities in Canadian populations, families and loved ones often live far from these facilities, sometimes even in other provinces. This context places the digital solutions as particularly relevant for the present COVID-19 pandemic as well as in the post–COVID-19 era.

**Objective:**

This project aims to co-develop a virtual community of practice through a web-based platform (eSocial-hub) to combat social isolation and loneliness among the older people in linguistic minority settings in Canada.

**Methods:**

An interventional study using a sequential mixed methods design will be conducted. Four purposely selected NFs will be included, 2 among facilities in Manitoba and 2 in New Brunswick; and 2 Anglophone NFs in Quebec will serve as knowledge users. The development of eSocial-hub will include an experimental 4-month phase involving the following end users: (1) older people (n=3 per NF), (2) families of the participating older people (n=3 per NF), and (3) frontline staff (nurse and health care aid; n=2 per NF).

**Results:**

Activities and solutions aiming at reducing social isolation and loneliness will be implemented and then evaluated with the project stakeholders, and the best practices generated. The assessment will be conducted using indicators derived from the 5 domains of the Consolidated Framework for Implementation Research. The project will be led by an interdisciplinary team and will involve a multisectoral partnership.

**Conclusions:**

The project will develop a promising and generalizable solution that uses virtual technology to help reduce social isolation and loneliness among the older people.

**International Registered Report Identifier (IRRID):**

PRR1-10.2196/30802

## Introduction

### Background

As vaccination for COVID-19 is ongoing worldwide, the third wave of the pandemic continues to surge. Canadian older adults in nursing facilities (NFs) paid the highest toll in terms of COVID-19 mortality, with 81% of deaths (first wave) versus 42% for all Organisation for Economic Co-operation and Development (OECD) countries [1]. This burden is compounded by the social isolation and loneliness endured by older people in NFs [2] as a consequence of public health measures that limit contact with professionals, family members, and caregivers. These unanticipated collateral effects increase vulnerability among older people [3] and continue to impact NF residents. This is particularly alarming in linguistic minorities, and poses a major challenge for managers and families, urging for innovative solutions—in this case, digital technologies. This approach is not only encouraged but also a necessity in light of its enormous potential to improve social capital and address the social isolation and loneliness of older people living in NFs. The literature contended that in addition to combatting social isolation and loneliness, video interactions increase learning effectiveness through the use of images [4], stimulate cognitive activity [5], and promote the transmission of knowledge [6].

### Burden of Social Isolation and Loneliness

Social isolation consists of reduced social contacts and loneliness (the subjective feeling of isolation); it represents a serious public health threat for older people [7] living in NFs. Social isolation and loneliness affect up to 72% of NF residents [8,9]; thus, there is a mounting concern in considering social isolation and loneliness as a determinant of the health and well-being among older people [10]. Owing to the *ageing tsunami* especially in high-income countries including Canada, costs of NFs and provision of care to older people are of the fastest-growing areas of governments’ spendings [11]. Additionally, the role of nurses, especially those working in NFs, has gained significant extension over the last decades [12] to deal with a broad range of care or services, namely (1) postacute care requiring rehabilitation and recovery, (2) terminal phases of an illness, or (3) management of (multiple) chronic conditions including cognitive or functional impairments [11]. Though the number of nurses in NFs has grown recently, evidence from a systematic review has unveiled nurse staffing issues in some of these facilities with nurses spending between 3.1 and 4.8 hours on each resident on ordinary days [11]. Undoubtedly, the COVID-19 pandemic has heightened this burden, and activities to counter social isolation and loneliness have taken a hit.

Besides the associated extra budget (eg, US $6.7 billion to Medicare costs [13]), social isolation and loneliness are associated with premature mortality [14], somatic diseases such as cardiovascular disease or obesity [15,16], or psychological issues including depression or anxiety [17]. The extreme vulnerability of older people is exacerbating the current COVID-19 crisis. For example, Francophone older people (≥65 years) in Manitoba are older than their Anglophone counterparts [18]. The majority of older people fear admission to NFs [19], some are either widowed, under guardianship, or have identified as sexual minorities (lesbian, gay, bisexual, transgender, questioning, or 2-spirited [LGBTQ2S+]) [20]. Furthermore, according to the Canadian Institute for Health Information, 87% of older adults in the country have some form of cognitive impairment, 69% have dementia, 50% experience behavioral problems, and 31% have depression [21]. As a previous study [11] highlighted that care outcomes targeted in NFs include changes and maintenance of a status and health condition–monitoring, with 2 unique dimensions of quality: quality of care and quality of life. Older people who are isolated or are experiencing cognitive decline may experience anxiety or behavioral problems (eg, agitation and withdrawal) during an outbreak or during lockdown [22]. Under normal circumstances, owing to the progressive collaborative culture, almost all (82%) older people in NFs benefit from their families’ involvement in visiting and in activities of daily living such as hygiene care and emotional and social support [21-23]. For instance, in Canada, at least 10 hours per week are devoted by one-fifth of families to their institutionalized loved ones [24]. Families’ role is of utmost importance in promoting and maintaining the social capital as the best source of ideas and knowledge [25] and for resolving social isolation and loneliness [26].

Apart from public health measures as one of the causes of social isolation and loneliness in older people, a dearth of health care workers over the successive waves contributes to the toll. Approximately 20% of Quebec’s health care workers were infected with COVID‑19 during the first wave [27]. NFs are understaffed and their exhausted personnel are living in fear owing to the high risk of becoming infected and infecting others. They operate under protocols that reduce previously observed physical interactions. Although NFs are a primary setting for recurrent disease outbreaks (eg, influenza), they are the least computerized segment of the Canadian health care [28]. While older people are often perceived as resistant to information and communication technology (ICT), surveys have noted a sharp increase (>40 per cent) in the use of ICT among this population since the 1990s [29].

### Social Isolation and Loneliness in Older Adults From Linguistic Minorities

As a numerical minority, the needs and realities of NFs for older adults from linguistic and cultural minority communities were given little consideration in the establishment of measures aimed at countering the effects of the COVID-19 pandemic. Additionally, older adults in linguistic NFs often live geographically far from their families, even in different jurisdictions. Francophones outside Quebec weight 3.5% and Anglophones in Quebec 7.5% [30]. Very few NFs serve older people who belong to these linguistic minority groups, and their residents generally live far from their families, sometimes even in other provinces. Compounding this issue, the COVID‑19 pandemic has revealed how public institutions and governments in multiple jurisdictions throughout Canada are failing to meet their linguistic obligations [31], which impedes timely access to information. Health systems did not demonstrate a strong surge capacity to address the pandemic of COVID-19 or its impact such as social isolation and loneliness, which could be more difficult for institutionalized older adults from linguistic and cultural minorities where even adults experience challenges in accessing the health care system [32]. The activities once offered by NFs to combat social isolation and loneliness (eg, outings) were even found to be effective [33], including the conventional ICT platforms (Skype, FaceTime, etc) currently offered by NFs to connect older adults with their families [34].

Nevertheless, the response to the pandemic (both the first wave and the third wave currently underway) has led to the successful implementation of some interventions (broadcasting video activities to patients in their rooms [35], video calling to bring together families and the institutionalized older people [36], and phone-based video calling, text messaging, or voicemail messaging [37]). Our project will capitalize on these practices, focusing on digital approaches to address social isolation and loneliness in anglophone minorities in Quebec and francophone minorities in NFs in Manitoba and New Brunswick.

Basically, our proposed web-based app, eSocial-hub, will allow interactive audio-video exchanges between the institutionalized older adults and their families. Some features such as autoresponse, à la carte ringing systems, and imaging pop off are intended to be tested. On the other hand, eSocial-hub will be designed to support socializing, fun, and educative activities.

### Purpose of the Project

This project aims to co-develop, implement, and assess a virtual hub (eSocial-hub), in partnership with end users at 4 NFs that serve Anglophone and Francophone older adults in minority settings. eSocial-hub will be a web-based digital platform, synchronized among the participating NFs to promote mainly the connection of institutionalized older people with their families and their frontline workers.

#### Objective 1

Our first objective is to identify and evaluate practices and lived experiences with families, residents, managers and frontline workers in using web-based apps to optimize older people's resilience and combat social isolation and loneliness during the COVID‑19 pandemic.

#### Objective 2

Our second objective is to co-develop eSocial-hub with older people, families, and professionals with a user-centered approach.

#### Objective 3

Our third objective is to implement and assess the community of practice’s eSocial-hub with older people, families, caregivers, and professionals in communicating and sharing resources (eg, entertainment activities, evidence-based findings, and chatting).

## Methods

### Methods Overview

This interventional study will be conducted in a minority community setting using a sequential mixed (qualitative/quantitative) design [38]. A collaborative approach will be used to involve end-users (managers, older adults, families, and frontline workers) in co-developing eSocial-hub. Four NFs in minority language/cultural settings, purposely selected, will be included: 1 in Manitoba (Résidence Despins) and 2 in New Brunswick (Manoir Edith B. Pinet Inc and Résidences Lucien Saindon). The 2 Anglophone NFs in Quebec (Jeffery Hale and Saint Brigid’s Home) will serve as knowledge users. The study will exclude older adults with terminal illnesses but will include those living with mild to moderate cognitive impairment.

### eSocial-hub Features

eSocial-hub includes voice/video calls, text messing, voice-mailing, and autoresponse features. Because it is developed for older people, a viable precaution has been made to eliminate any technicality and provide an information technology (IT)–lay environment that usually hinders IT product use. eSocial-hub will support families and their institutionalized loved ones in their daily communication needs, prompted either by the former or the latter or by frontline workers. eSocial-hub is designed to be interoperable between exploitation systems and uploadable on cell phones, tablet devices, as well as laptops. The platform will be host by the University of Ottawa’s IT infrastructure with biweekly maintenance by its IT’s staff.

As a communication based-system, eSocial-hub is an internet-based app that adds to the running cost paid by the project budget. The study team is distinct with the developers of the platform as well as the funder. Its role toward the system being evaluated is to inform the developer on the expected features and finally evaluate its effectiveness. The project will be carried out in 3 phases.

### Inventory of Interventions in Digital Technologies (Phase 1: June 1 to July 30, 2021)

This phase will involve several steps. During step 1, an inventory of interventions that use digital technologies to reduce the harms of isolation and loneliness in residents during the COVID‑19 pandemic will be conducted. The study sample will include participants in the NFs as well as families. Therefore, we will select the chief executive officer and 2 other major program managers (nursing chief and social worker), 3 in each NF (n=12) for a semistructured interviews. We also plan to select frontline workers including nurses (n=12) and health care aides (n=12). Step 2 will explore lived experiences in maintaining social connections and social capital between families and their older residents. Thus, 1 focus group per site (n=4), completed with a short structured survey of families (n=80), will be performed through Lime Survey. Using data collected in Phase 1, a deliberative workshop will then be held with stakeholders (4 older adults, 4 families/caregivers, 4 managers, and 4 frontline staff) to validate appropriate strategies, implementation modalities, and success indicators on the basis of the 5 domains of the Consolidated Framework for Implementation Research (CFIR) [39] (Phase 3), integrating sex, gender, and LGBTQ2S+ [40], social and linguistic justice, and cultural and racial diversity factors. The study will include participants from these diverse groups as much as possible; alternatively, at least half the participants will be men and the other half will be women. Activities will take place in both languages: French for older people in Francophone NFs and English for those residing in Quebec NFs.

### eSocial-hub Co-Development Trial (Phase 2: August 5 to October 30, 2021)

Participants who consent to continue with Phase 2 will be asked to renew their written consent. For new participants, written consent will be solicited. Based on feedback from the participating NF concerning their experiences with digital technology, the co-development team will build eSocial-hub to share promising evidence-based practices. One day will be devoted to train the participants to use the platform. This process will take place during a 3-month pilot phase. Consenting families and their loved older adults will be involved in an experimental group comprising 2 expert older adults per NF (n=8), 2 families or caregivers per NF (n=8), and 1 frontline worker (1 health care aid or 1 nurse) per NF (n=4). They will use a device (tablet, laptop or desktop computer) connected to the internet. In a departure from traditional approaches, testing of eSocial-hub will be conducted using a virtual *Hackerspace* to complement the data collected from the experimental group. *Hackerspace* will be a chat platform open to friends of older people, NF users, older people's associations, and interested members of the public.

**Figure 1:**
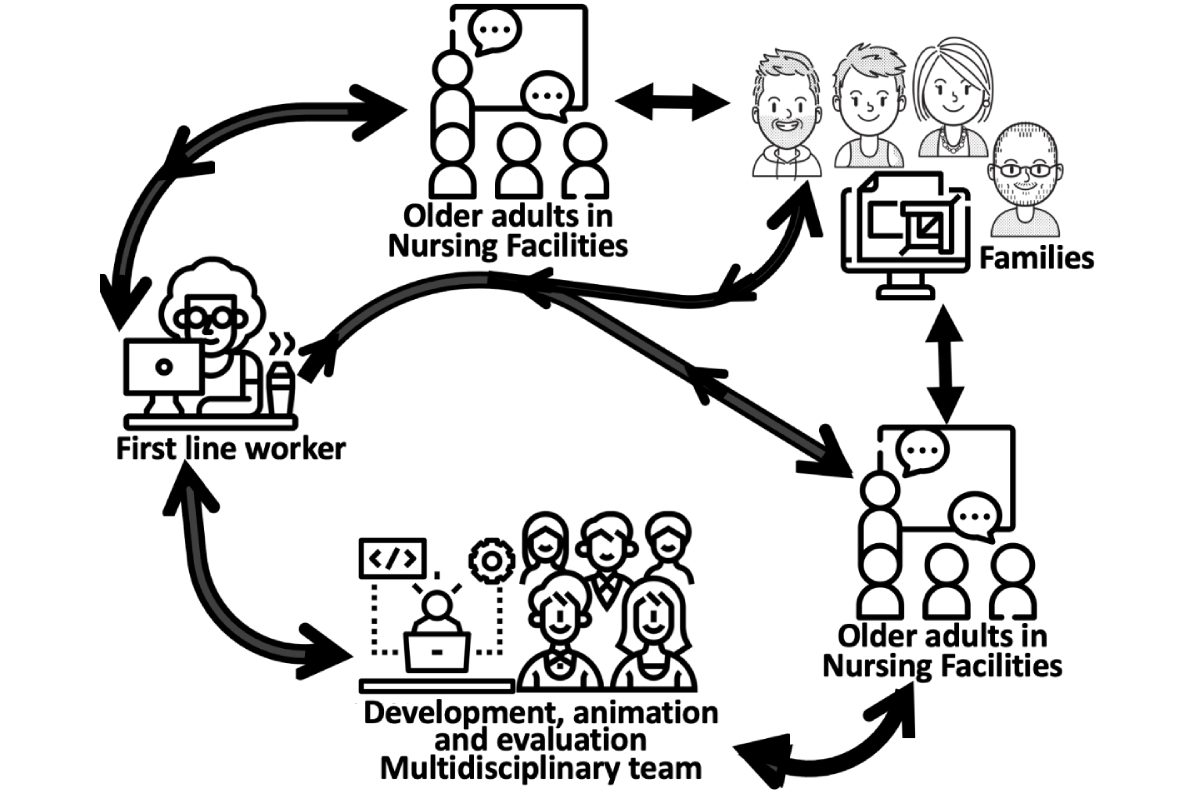
Modus operandi of the eSocial-hub.

The deliberative workshop and the interviews will be conducted entirely on the internet. eSocial-hub will be designed and validated by a software developer, following the DMAIC (Define, Measure, Analyze, Improve, and Control) approach [41]. The DMAIC cycle will optimize the efficiency of eSocial-hub, improve its user-friendliness, and enhance its features’ functionality on the basis of the modus operandi of eSocial-hub (Figure 1). The following indicators will be measured: results optimization, aesthetics, ergonomics, operational reliability, and durability [42]. User feedback (complaints, comments, and observations) will be compiled consecutively during this trial in the form of text messages and audio messages. These will be analyzed biweekly with personalized follow-ups by the research team, as needed.

### Content of eSocial-hub

This will be defined by the deliberative workshop. However, in addition to the chat and audiovisual features used to connect older people and their families or frontline workers (or to connect 3 types of users simultaneously), the eSocial-hub will provide an outlet to share activities in real time. Furthermore, entertainment activities will be offered by several nursing students who will be trained in audiovisual facilitation/presentation by our partner, La Liberté, Manitoba’s sole French-language newspaper. Students’ participation in the project will allow them to achieve their personal and professional development objectives.

### eSocial-hub Trial Assessment Strategy (Phase 3: October 10 to November 5, 2021)

**Figure 2:**
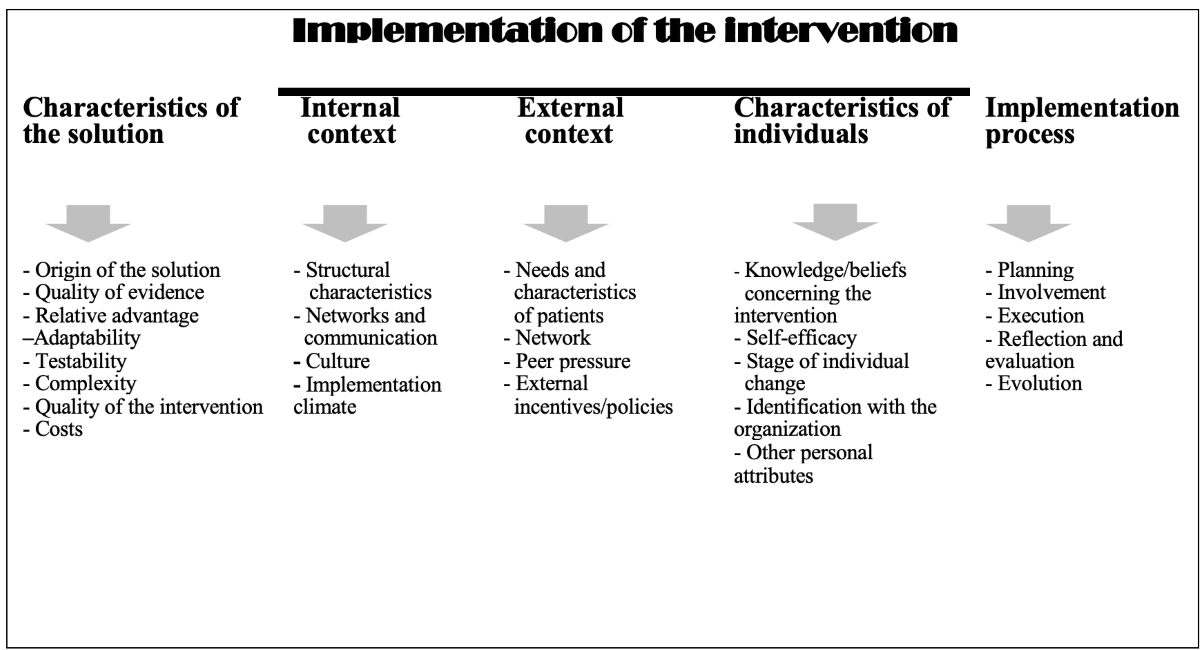
Main components of the CFIR and corresponding constructs (adapted from [44]).

The assessment will analyze indicators defined by consensus during the deliberative workshop, in accordance with the 5 CFIR domains (Figure 2), and integrate patient-partner and diversity factors (cultural, sex, gender, and LGBTQ2S+, and linguistic justice). The CFIR’s metatheoretical framework draws on concepts from multiple theories and models [39] based on expert consensus. It includes five components: (1) the characteristics of the intervention, (2) the external context, (3) the internal context, (4) the characteristics of the individuals, and (5) the implementation process. Figure 2 presents the various constructs and factors that will be considered for each component to optimize and evaluate the eSocial-hub’s implementation. We will use the indicators to develop a structured survey questionnaire, using a 6-point Likert scale (to prevent an average response bias [43]). The questionnaire will be administered before and after the trial.

The final questionnaire items will be validated through a modified Delphi process [45]. Six end-users (3 families and 3 older adults) and 5 experts will be consulted to obtain a consensus on the indicators to be considered. The survey will consist of two sections: the first set of questions on the personal and professional characteristics of the respondents (age group, gender, role, type of organization, time in the organization, involvement in the intervention) and a second section on the proposed CFIR indicators. For each of the indicators, the respondents will have to indicate its degree of importance for the implementation of the innovation on a 6-point Likert scale (1=not important to 6=very important). Analyses will be performed to calculate the median and interquartile range for each indicator. Indicators with a score of 5 or 6 and an interquartile range of ≤1 will be retained as consensus. Indicators with a score of 4 or less and an interquartile range of ≤1 will be excluded. The second round of surveys will be conducted with the same respondents to assess indicators that did not have a consensus for retention or exclusion in the first round. For this second survey, the score given by the respondent in the first round as well as the median score obtained will be presented, and then the respondent will be asked to change their score if they wish to reach a consensus. If there is no consensus on certain indicators following this second round, a third round may be conducted. Respondents will have 1 week to complete each round of the survey and a reminder will be sent to them after 3 days.

Finally, using a utilization-focused evaluation approach [46], our team will coordinate the monitoring of the trial phase at mid-project (August 2021) and at the end of the project (December 2021) and conduct follow-up interviews with the trial participants to further explore some of the quantitative results. The use of mixed methods will allow for a more detailed analysis of the implementation process, taking into account the local context and dynamics. The findings will be shared during a web-based meeting with the NF teams, and their feedback will be incorporated into the final evaluation report and a peer-reviewed paper presenting the results. The complementary expertise of the team members in implementation science and evaluation, digital technologies (MPG), nursing and long-term care (IB), public health (DS), psychology (JR and NJCB), health economics (ENT), sociology and health services organization (AA), and organizational studies (SC) will enrich the analyses from multiple perspectives. This cohesive research team has been involved in research collaborations since 2015 and completed and published numerous projects [47-55]. Much of their recent work [52,54,55] is closely related to this project.

### Data Analysis

The quantitative analysis will be descriptive. Data will also be subjected to a bivariate analysis (analysis of variance or *t* test, as appropriate). In the event of a non-Gaussian distribution, the Mann–Whitney test will be used, with *P*<.05 considered significant. The qualitative analysis will involve data from focus groups and individual interviews. Verbatims will be transcribed and imported into N-Vivo 12 analysis software. Inductive thematic analysis will be carried out by at least 2 coresearchers independently working to develop preliminary coding structures to organize the data thematically (IB and JR) [56] to understand the meaning of the participants’ experience [57]. Coding in 2 phases will refine the relationship among categories to be explored to facilitate the raising of the analytical level from categorical to thematic for meaningful interpretations of the data. Emerging themes will be defined by consensus by the research team [58]. Rigor credibility will be achieved by obtaining data from all stakeholders and investigator triangulation [59]. For its multi-site feature, we intend to achieve the transferability of the findings by providing a clear description of the participants, settings, and research process [60]. We will achieve confirmability through data triangulation and researcher reflexivity [59]. Finally, quantitative and qualitative data will be triangulated [61].

## Results

This study was funded in April 2021. We plan to start active enrollment, and data collection will start on June 1, 2021. The project was granted ethical approval from the ethical committee for research of the University of Saint-Boniface and the University of Moncton (2021-085). This project is intended to end by February 2022. As of December 2021, we will have concluded the project evaluation. Early winter 2022 is the anticipated period to disseminate nationally and internationally the results generated.

## Discussion

Social isolation and loneliness are prominent topical issues since the onset of the first wave of the COVID-19 pandemic. Best practices for the use of web-based apps will optimize institutionalized resilience among older people in combatting isolation and loneliness. Furthermore, the development of eSocial-hub using a user-centered approach is innovative to prove a concept and pave the way for health policymakers based on its added value, namely for older people in the context of the linguistic minority.

This project stems directly from a need felt and expressed by the participating NFs that are partnering with the 3 universities involved in this project. It is clear that NFs have been overwhelmed by the effects of COVID‑19. They remain a weak link in our system, subject to recurring outbreaks (eg, seasonal illnesses). We expect to validate the eSocial-hub concept as a means to combat social isolation and loneliness. This issue will persist in the post–COVID‑19 era, considering that NF may find it necessary to continue imposing certain restrictions, namely physical access to NF. This ensures the pertinence of a virtual solution. During eSocial-hub’s testing phase, the expected outcomes include, in addition to the objective of combatting social isolation and loneliness, the use of digital tools (eg, iPad and laptop computer) that subsequently help stimulate and develop learning and cognitive activity in older adults. eSocial-hub, in addition to connecting families and caregivers with their older members, will allow for secure communications with staff, including live interactions and support a web-based professional community of practice as well as services and entertainment activities that were previously offered in person (eg, religious services, music, entertainment, lectures, and bingo sessions).

Inaugural entertainment activities will be offered by nursing students of the Université de Saint-Boniface after receiving training in facilitating and presenting audiovisual media from our partner, the La Liberté newspaper (the only French-language publication in Manitoba).

The results of this project will be published in a peer-reviewed open-access journal for public dissemination. We intend to participate in at least 1 colloquium or conference. These knowledge dissemination activities will raise awareness among co-researchers and decision-makers concerning the positive effects of the web-based hub on social isolation and loneliness (and therefore the quality of life) among older people in linguistic and cultural minority communities in the context of the COVID‑19 pandemic.

The anticipated potential challenges pertain to the limited availability of managers and frontline staff in the event of COVID‑19 outbreaks.

## Abbreviations

CFIR: Consolidated Framework for Implementation Research
DMAIC: Define, Measure, Analyze, Improve, and Control
ICT: information and communication technology
NF: nursing facility
OECD: Organisation for Economic Co-operation and Development
